# The Preservation of the Therapeutic Efficacy of the Secretome of Adipose Mesenchymal Stem Cells, Produced in the Presence of Antioxidant and Anti-Inflammatory Drugs

**DOI:** 10.3390/pharmaceutics17091171

**Published:** 2025-09-08

**Authors:** Sofia Martinez-Rodriguez, Nahla Jemni-Damer, Atocha Guedan-Duran, Girish K. Srivastava, Fivos Panetsos

**Affiliations:** 1 Departamento de Cirugía, Oftalmología, Otorrinolaringología y Fisioterapia, Facultad de Medicina, Universidad de Valladolid, 47005 Valladolid, Spain; sofia.martinez.rodriguez22@uva.es (S.M.-R.); girishkumar.srivastava@uva.es (G.K.S.); 2 Instituto Universitario de Oftalmobiología Aplicada, Facultad de Medicina, Universidad de Valladolid, 47011 Valladolid, Spain; 3 Silk Biomed SL, 28049 Madrid, Spain; njemni@silkbiomed.com; 4 Bioactive Surfaces SL, 28049 Madrid, Spain; aguedan@bioactivesurfaces.com; 5 Neurocomputing and Neurorobotics Research Group, Faculty of Biology and Faculty of Optics, Universidad Complutense de Madrid, 28040 Madrid, Spain; 6 Institute for Health Research San Carlos Clinical Hospital (IdISSC), 28040 Madrid, Spain; 7 Omnia Mater SL, 28049 Madrid, Spain; 8 Human Retina SL, 28009 Madrid, Spain

**Keywords:** mesenchymal stem cells, secretome profiling, drug exposure, anti-inflammatory drug, retina inflammation

## Abstract

**Background/Objectives:** Inflammatory processes, both acute and chronic, encompass a wide range of autoimmune, metabolic, and neurodegenerative conditions. Conventional treatments, primarily anti-inflammatories and immunosuppressants, provide partial relief but are often hampered by adverse effects and limited durability. Mesenchymal stem cells (MSCs) have emerged as a powerful new treatment due to their immunomodulatory and anti-inflammatory properties, primarily mediated through their secretome, which is a complex mixture of bioactive factors. Secretome-based therapeutic strategies show strong potential for controlling inflammation, mitigating oxidative stress, and supporting tissue regeneration and repair. However, the therapeutic efficacy of MSCs’ secretome is subject to modification by concurrent anti-inflammatory drug regimens used in clinical settings. **Methods:** To evaluate the effect of combinatorial treatment strategies on the secretome of the MSCs, we employed an in vitro retinal inflammation model to investigate whether the exposure of the MSCs to five representative anti-inflammatory drugs (ketorolac, diclofenac, α-lipoic acid, N-acetyl-L-cysteine, and nicotinamide) impacts the functionality of the resulting secretome. Specifically, we evaluated the effect of the above-mentioned drugs on the anti-inflammatory properties of the secretome in relation to the secreted levels of two main MSC secretome factors—the Brain-Derived Neurotrophic Factor (BDNF) and the Vascular Endothelial Growth Factor (VEGF)—and on the secretome’s pro-metabolic activity. **Results:** Our findings provide evidence that the presence of any of the tested drugs during MSC secretome production does not compromise its anti-inflammatory activity; BDNF and VEGF levels remain stable, and the secretome retains a high degree of its pro-metabolic effect. **Conclusions:** These results underscore the robustness and clinical resilience of MSC-based therapies, even when administered alongside pharmacological agents. This work advances the translational viability of MSC therapies for inflammatory diseases and supports the development of safe, combinatorial treatment strategies.

## 1. Introduction

Inflammatory conditions, both chronic and acute, represent a significant global health challenge due to their multifactorial nature and persistent immune dysregulation [1]. These disorders include autoimmune conditions such as rheumatoid arthritis, chronic inflammatory diseases like inflammatory bowel disease (IBD), and non-autoimmune conditions with strong inflammatory components such as type 2 diabetes [2]. Their common denominator is sustained inflammation, which drives tissue damage, organ dysfunction, and progressive deterioration of quality of life [3].

### 1.1. Inflammatory Pathways

The most critical pro-inflammatory pathways are those that integrate danger sensing with the transcription and release of inflammatory mediators. The NF-κB pathway (nuclear factor kappa-light-chain-enhancer of activated B cells) acts as a central regulator of immune and inflammatory responses [4]. It is activated by receptors such as cytokine receptors, pattern-recognition receptors (PRRs), TNF receptor (TNFR) superfamily members, and T-cell receptors (TCRs) and B-cell receptors. Once activated, NF-κB translocates to the nucleus, inducing genes that encode cytokines, chemokines, adhesion molecules, and inflammasome components [5,6].

Among these components, the NLRP3 inflammasome (NOD-like receptor protein 3) is particularly important [7]. It requires an initial step of priming, often provided by NF-κB signaling, to increase the expression of NLRP3 and pro-forms of IL-1β. A second step of activation is then triggered by stimuli such as extracellular ATP, mitochondrial dysfunction, crystalline particles, or changes in ion fluxes [8]. Once activated, NLRP3 recruits and activates caspase-1, which processes pro-IL-1β and pro-IL-18 into their mature, bioactive forms, as well as inducing pyroptosis, which is a lytic form of programmed cell death that releases additional danger signals [9].

In parallel, MAPK cascades (mitogen-activated protein kinases), including ERK (extracellular signal-regulated kinase), JNK (c-Jun N-terminal kinase), and p38, transmit stress and inflammatory signals to the nucleus, where they activate transcription factors such as AP-1 (activator protein 1) [10]. These cascades not only promote the expression of inflammatory genes but also cross-talk with NF-κB and NLRP3 pathways, amplifying the overall inflammatory response [11].

Finally, ROS (reactive oxygen species), generated by enzymes like NADPH oxidases or during mitochondrial stress, serve a dual role—they cause oxidative damage to lipids, proteins, and nucleic acids, as well as acting as secondary messengers that further activate NF-κB, MAPK, and NLRP3 inflammasome signaling [12].

Together, these pathways, NF-κB, NLRP3 inflammasome, MAPK cascades, TLR signaling, and ROS production form an interconnected network. This network creates positive feedback loops that are highly effective in mounting acute inflammation [13].

### 1.2. Anti-Inflammatory Treatments

Despite growing insights into their pathogenesis, the therapeutic options for many inflammatory diseases remain largely palliative. Current clinical strategies typically involve immunosuppressants, corticosteroids, or biologics targeting specific cytokines or immune pathways [14]. Although these agents provide symptomatic relief, their long-term use is frequently limited by diminishing efficacy and adverse side effects. Moreover, some patients experience intolerance or allergic reactions to these treatments, further restricting their clinical utility [15]. This underscores the urgent need for safer, more effective therapies that not only alleviate symptoms but also target the underlying inflammatory mechanisms in a more personalized and tolerable manner.

In this context, mesenchymal stem cells (MSCs) have gained attention as a promising new anti-inflammatory treatment [16]. These multipotent stromal cells, present in several tissues such as bone marrow, adipose tissue, or umbilical cord, exert profound anti-inflammatory effects, largely mediated through the secretion of bioactive molecules, collectively referred to as the secretome. Their secretome includes cytokines, chemokines, growth factors, and extracellular vesicles that regulate immune responses, reduce oxidative stress, promote angiogenesis, and facilitate tissue repair [17]. The application of MSCs in the treatment of inflammatory diseases has become particularly prominent due to their ability to target multiple aspects of the inflammatory cascade [18]. Unlike conventional anti-inflammatory drugs that often act on single pathways and may lead to adverse effects with long-term use [19], MSCs exert a broad immunomodulatory effect, influencing both innate and adaptive immune responses [20]. This makes them suitable for treating a wide range of chronic inflammatory conditions, such as rheumatoid arthritis [21], inflammatory bowel disease [22], and multiple sclerosis [23], among others. The growing number of clinical trials exploring MSC-based therapies in these contexts underscores their potential to become a cornerstone in next-generation treatment strategies [24].

Due to their low immunogenicity, MSCs are suitable for both autologous and allogenic implants [25]. However, cell transplantation faces several challenges, including the potential alteration of cells’ secretome, which may be influenced by the characteristics of the surrounding microenvironment [26]. In this context, it is important to understand how MSCs’ secretome could also be conditioned by drugs that are commonly used in clinical practice [27], since patients undergoing MSC-based treatments could simultaneously receive pharmacological therapies; such interactions may potentially alter the secretome’s therapeutic efficacy [28]. However, despite the importance of how drug exposure affects the MSC secretome and how it could modify its functional properties, very few studies have addressed this problem [29].

Understanding these interactions is particularly critical for inflammatory diseases, where the precise modulation of immune activity and oxidative stress is essential for controlling pathology and promoting repair [30]. In the present paper, we investigate whether the exposure of MSCs to commonly used anti-inflammatory and antioxidant drugs alters the composition and therapeutic function of the MSC-derived secretome and we also evaluate whether the impact of clinically relevant anti-inflammatory drugs enhances, impairs, or preserves the ability of the MSC secretome to promote cell survival and regulate inflammation in an in vitro inflammatory model.

To this end, we selected five representative compounds with known anti-inflammatory or redox-regulatory activity—ketorolac and diclofenac (NSAIDs that inhibit cyclooxygenase activity and prostaglandin synthesis) [31], α-lipoic acid and N-acetyl-L-cysteine (antioxidants that neutralize reactive oxygen species (ROS)) [32,33], and nicotinamide, which is a precursor of NAD^+^ that supports redox homeostasis via NADPH generation [34]. Although widely used in inflammatory and degenerative contexts, their influence on MSC secretome composition and the function of the secretome remains poorly defined [34]. Our study focuses on determining whether the presence of clinically relevant anti-inflammatory drugs alters the level of key secreted factors, e.g., Brain-Derived Neurotrophic Factor (BDNF) and Vascular Endothelial Growth Factor (VEGF), or whether it affects the anti-inflammatory activity and metabolic support of the surrounding cells.

The drugs under consideration in the present paper act on precise points of the above inflammatory pathways. On the one hand, ketorolac and diclofenac inhibit COX-1/COX-2, reducing prostaglandin synthesis and inflammation; this also downregulates the NF-κB and MAPK pathways, decreasing COX-2 expression and pro-inflammatory cytokines, and indirectly limits NLRP3 inflammasome activation [32,35]. On the other hand, α-Lipoic acid (ALA) and N-acetylcysteine (NAC) reduce oxidative stress by scavenging ROS and restoring glutathione levels, which suppresses NF-κB, MAPK, and NLRP3 activation. This leads to decreased COX-2 and pro-inflammatory cytokines, providing strong antioxidant and anti-inflammatory effects [31,33,36]. Finally, nicotinamide, which is a precursor of NAD^+^ that supports redox homeostasis via NADPH generation, lowers oxidative stress and reduces the activation of NF-κB, MAPK, and NLRP3, which decreases pro-inflammatory mediators [34,37].

BDNF and VEGF are critical factors secreted by MSCs that play essential roles in modulating and resolving inflammation. BDNF suppresses the expression of pro-inflammatory cytokines such as TNF-α, IL-1β, and IL-6; enhances anti-inflammatory signaling pathways; and mitigates secondary tissue damage [38]. VEGF, beyond its angiogenic function, facilitates macrophage polarization toward the M2 phenotype, thereby promoting an anti-inflammatory environment and supporting tissue repair [39].

## 2. Materials and Methods

**Figure 1:**
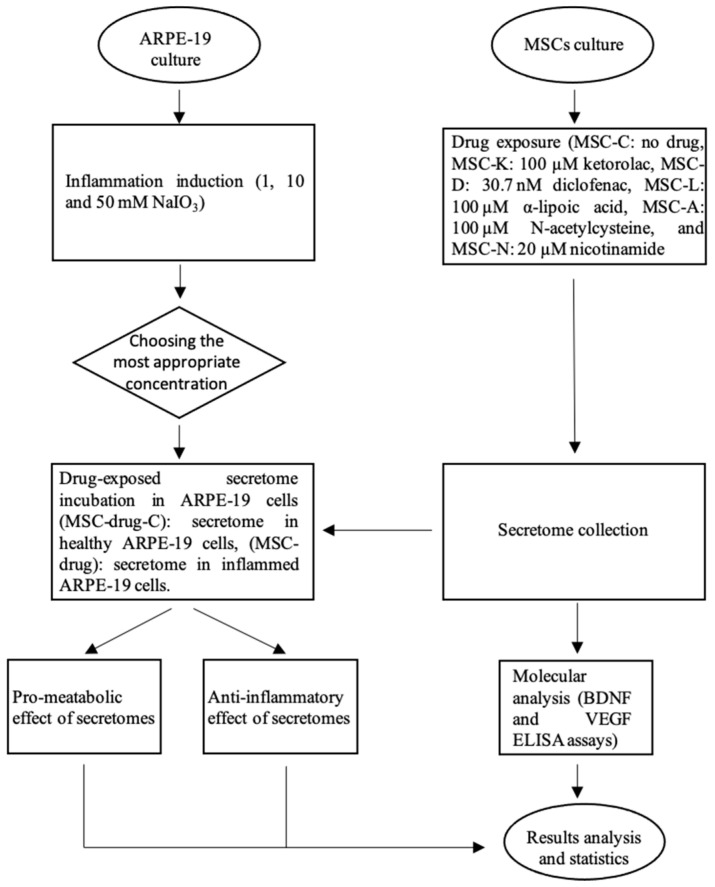
Overview of the experimental workflow. The flow chart summarizes the methodology used in this study, including cell culture preparation, the induction of the inflammatory model, treatment with selected compounds, sample collection, and subsequent analyses.

To investigate whether the exposure of MSCs to commonly used anti-inflammatory and antioxidant drugs alters the composition and therapeutic function of the MSC-derived secretome, we applied the following experimental procedure (Figure 1).

### 2.1. MSC-Derived Secretome

#### 2.1.1. Cell Cultures

Adipose-derived human MSCs (StemPro^®^ Human Adipose-Derived Stem Cells) were obtained from Invitrogen™ (Carlsbad, CA, USA). These cells were isolated from the lipoaspirate tissue of a single donor, expanded through one passage, and then cryopreserved. The supplier validated their characteristics through a flow cytometry analysis of their cell surface protein profile. The MSCs were cultured under standard conditions at 37 °C and 5% CO_2_, adhering to the surface of 100 mm culture plates (Fisher Scientific, Cat. No. 11815275, Waltham, MA, USA) with complete DMEM low [Glu] medium (Fisher Scientific, Cat. No 11564446) supplemented with 10% Fetal Bovine Serum (Fisher Scientific, Cat. No. 17479633), 1% Penicillin/Streptomycin (Fisher Scientific, Cat. No 11548876), and 1% L-glutamine (Fisher Scientific, Cat. No. 11500626) until reaching 70–80% confluence. At this point, the adherent cells were detached using 0.05% Trypsin-EDTA (Fisher Scientific, Cat. No. 11590626) and were reseeded into 24-well culture plates (Fisher Scientific, Cat. No. 10604903) to subsequently be used for secretome production. For the cell inflammation model, human retinal pigment epithelial cells (ARPE-19 cell line) [American Type Culture Collection^®^ (ATCC^®^), Manassas, VA, USA] were used. The cells were cultured in complete DMEM/F12 medium (Merck Cat. No. D8437, Darmstadt, Germany) supplemented with 10% Fetal Bovine Serum and 1% Penicillin/Streptomycin and were maintained under standard conditions in 100 mm culture plates until reaching 90–95% confluence. Then, the cells were detached using 0.05% Trypsin-EDTA and were reseeded into 24-well culture plates.

#### 2.1.2. MSC Secretome Production

To assess whether different drugs influence the molecular content and the effectiveness of the MSC secretome, MSCs were exposed to the five commercially available anti-inflammatory compounds. The cells were divided into one control (MSC-C: control—MSCs without any drug) and five experimental groups. The experimental groups were as follows: MSC-K: MSCs with 100 µM ketorolac (Sigma-Aldrich, Cat. No. K1136-1G, St. Louis, MO, USA); MSC-D: MSCs with 30.7 nM diclofenac (Sigma-Aldrich, Cat. No. D6899-10G); MSC-L: MSCs with 100 µM alpha-lipoic acid (Sigma-Aldrich, Cat. No. T5625-1G); MSC-A: MSCs with 100 µM acetylcysteine (Sigma-Aldrich, Cat. No. A7250-5G); and MSC-N: MSCs with 20 M nicotinamide (Sigma-Aldrich, Cat. No. N0636). MSCs were treated with the corresponding drug for 24 h. Afterwards, 5 × 10^4^ cells were seeded in 24-well plates and were cultured in specific cell medium. When the cells reached 70% to 80% confluence, the medium was replaced with a fresh one and the respective drugs were added; the cells were left for 24 h. Then, the culture supernatant was collected and saved for subsequent testing.

The concentrations employed in our study were selected based on evidence from previous reports in the literature. Several independent studies have demonstrated that these concentrations produce consistent and biologically relevant effects in the corresponding experimental models without causing cytotoxicity [40,41,42,43,44,45,46,47,48,49]. We therefore adopted these values to ensure comparability with prior work and to build upon established findings.

#### 2.1.3. MSC Secretome Evaluation

To investigate whether the administration of different drugs influences BDNF and VEGF secretion by MSCs, two ELISA assays were performed (Fisher Scientific, Cat. No. 17164733 and Fisher Scientific, Cat. No. KHGO111). The protocols provided by the manufacturer were followed. Briefly, 50 µL of culture supernatant samples were introduced to wells that were pre-coated with the specific antibodies. Plates were incubated for 2 h at 4 °C with gentle shaking. After that, four washes were performed and a biotin-conjugated secondary antibody was added. Following a 1 h incubation, another four washes were carried out. Streptavidin conjugated to an enzyme was then added and incubated for 30 min at room temperature, protected from light. Finally, the enzyme substrate was added and absorbance was immediately measured at 450 nm using a microplate reader (ELx800, BioTek, Winooski, VT, USA).

### 2.2. Set-Up and Optimization of an In Vitro Cell Inflammation Model

#### 2.2.1. Induction of Cell Inflammation by NaIO_3_

To study the effect of the drugs on the secretome produced by the MSCs in the presence of anti-inflammatory drugs, we had to test the efficiency of such a secretome in regulating an inflammatory process, such as, for example, in age-related macular degeneration or in inflammatory bowel diseases. For this reason, we set up an in vitro model of retinal inflammatory conditions based on NaIO_3_ treatment [50]. ARPE-19 cells were treated with different concentrations of NaIO_3_ to maximize the inflammatory response, while minimizing the impact of NaIO_3_ on cell viability. The ARPE-19 cells were cultured in specific medium and were maintained under standard conditions in 100 mm tissue culture dishes until reaching 90–95% confluence. Then, 5 × 10^4^ cells were seeded in 24-well plates. Furthermore, 24 h later, when the cells reached 80% confluence, NaIO_3_ (Sigma-Aldrich, Cat. No S4007) was added at the following concentrations: 1 mM, 10 mM, and 50 mM for 24 h. After that, cell inflammation and cell metabolic rate were studied. As an inflammation biomarker, the nitric oxide (NO) released by the damaged cells into the culture medium was employed, while the Griess reagent was used to quantitatively assess these NO concentrations [51,52]. As a metabolic rate biomarker, XTT reagent (2,3-bis(2-methoxy-4-nitro-5-sulfophenyl)-5-[carbonyl(phenylamino)]-2H-tetrazolium hydroxide) was used, as its reduction by mitochondrial enzymes in viable cells produces a measurable color change that reflects metabolic activity [53].

#### 2.2.2. Measuring NaIO_3_-Induced Inflammatory Response In Vitro

The Griess reagent was prepared as follows: a 1% (*w*/*v*, 10 mg/mL) solution of sulfanilamide (Sigma-Aldrich, Cat. No. S9251) was dissolved in 5% phosphoric acid (Sigma-Aldrich, Cat. No. 345245) (*v*/*v*) and a 0.1% solution of NED (*N*-(1-Naphthyl) Ethylenediamine dihydrochloride) (Sigma-Aldrich, Cat. No. 222488) was dissolved in distilled water (1 mg/mL). The two components were prepared separately and mixed in a 1:1 ratio immediately before use. One day post-treatment of the ARPE-19 cells with the different concentrations of NaIO_3_, a 100 µL sample from each well was collected and mixed with an equal volume (100 µL) of Griess reagent. Simultaneously, a NaNO_2_ (Sigma-Aldrich, Cat. No. 31443) 1 mM solution was prepared in the specific ARPE-19 medium for the standard curve. Serial dilutions were made from 100 µM to 1.56 µM; absorbance was measured alongside the samples. The absorbance was immediately read at 540 nm using a microplate reader (ELx800, BioTek).

#### 2.2.3. Measuring Cell Metabolic Rate Following NaIO_3_-Induced Damage In Vitro

ARPE-19 viability test after treatment with the different secretomes was performed by means of an XTT assay, measuring cellular metabolic activity through the enzymatic reduction of tetrazolium salts [54].

To evaluate the impact of the different concentrations of NaIO_3_ on the metabolic rate of ARPE-19 cells, absorbance was measured using an XTT assay kit (Fisher Scientific, Cat. No. 15960972). For the assay, a mix of 70 µL XTT solution (prepared by combining 7 mL of XTT reagent with 1 mL of electron coupling reagent) was added to 170 µL of cell culture medium per well. The culture medium was removed and replaced with fresh medium containing the XTT mixture. The cells were incubated for 4 h at 37 °C with 5% CO_2_. After incubation, absorbance was measured at 450 nm using a microplate reader (ELx800, BioTek).

Optimal effects were obtained with 10 mM NaIO_3_ treatment during 24 h (see Results, Section 3.1—NaIO_3_-induced cell inflammation).

### 2.3. Effect of Anti-Inflammatory Drugs on the Efficacy of the MSC Secretome

The drugs’ impact on the anti-inflammatory efficacy of the MSC secretome was evaluated by treating the 10 mM NaIO_3_-damaged ARPE-19 cells with secretomes produced in the presence of each of the anti-inflammatory drugs. NO levels were measured one day after treatment using the Griess reagent, following the previously described protocol. Similarly, the drugs’ impact on the cell metabolic rate was evaluated by measuring absorbance one day post-treatment of the 10 mM NaIO_3_-damaged ARPE-19 cells using the above XTT test.

### 2.4. Statistical Analysis

Statistical analyses were conducted using IBM SPSS Statistics (version 26). The normality of the data was assessed using the Shapiro–Wilk test. For parametric data, i.e., datasets that followed a normal distribution (*p* > 0.05, Shapiro–Wilk test), one-way ANOVA was performed to compare means across multiple groups. When statistically significant differences were detected (*p* < 0.05), Tukey’s post hoc test was used to determine pairwise group differences. Data are presented as mean ± standard deviation (SD). For non-parametric data, i.e., datasets that did not follow a normal distribution (*p* < 0.05, Shapiro–Wilk test), the Kruskal–Wallis test was used to assess differences among groups. In cases where the Kruskal–Wallis test yielded significant results, pairwise comparisons were performed using the Mann–Whitney U test with Bonferroni correction for multiple comparisons. When no significant differences were found in the overall test (*p* > 0.05), no post hoc analysis was conducted. Data in non-parametric analyses are presented as median with interquartile range (M (IQR)). In all cases, *n* = 4 and statistical significance was considered at *p* < 0.05.

## 3. Results

### 3.1. NaIO_3_-Induced Cell Inflammation

To optimize the appropriate concentration of NaIO_3_ for the establishment of a retinal inflammation model, different concentrations of NaIO_3_ were tested (1, 10, and 50 mM) to identify the concentration that induces the strongest inflammatory response with a minimal effect on cell metabolic rate, providing a platform to study the reparative effects of the MSC-derived secretome. After 24 h of exposure to NaIO_3_, the NO levels released into the supernatant, as well as the cell metabolic rate, were measured.

**Figure 2:**
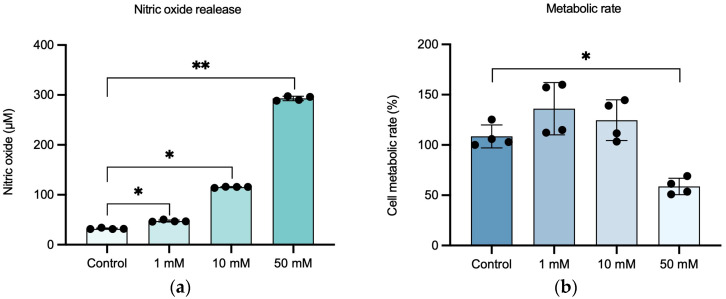
Set-up of the in vitro retinal inflammation model on ARPE-19 cells damaged with NaIO_3_ concentrations of 1, 10, and 50 mM. (**a**) Relationship between NaIO_3_ concentration and NO release of ARPE-19 cells. (**b**) Effect of NaIO_3_ concentration on the functionality (metabolic rate) of ARPE-19 cells after 24 h. Statistically significant differences were observed between control and inflammatory conditions in NO release, as well as between control and 50 mM inflammatory conditions in metabolic rate. Data in (**a**) are shown as M (IQR); data in (**b**) are shown as μ ± SD. Dots in bars represent each replicate. * *p*-value < 0.05; ** *p*-value < 0.01.

Treatment of ARPE-19 cells with increasing concentrations of NaIO_3_ resulted in a significant elevation of NO levels compared to the untreated control group (Figure 2a). M (IQR) levels of NO were 31.67 (2.04), 46.67 (3.05), and 115.9 (1.9) in the 1, 10, and 10 mM groups, respectively. The highest response was observed in the 50 mM group, with an M (IQR) of 293.1 (8.2).

In terms of cell metabolic rate (Figure 2b), no statistically significant reduction was observed in the groups treated with 1 and 10 mM NaIO_3_ (136.1 ± 26.01 and 124.6 ± 20.26, respectively) compared to the control group (108.6 ± 11.41), while exposure to 50 mM NaIO_3_ led to a marked and statistically significant decrease in cell metabolic rate (58.74 ± 8.23). *p* < 0.05.

### 3.2. The Presence of Anti-Inflammatory Drugs Does Not Impair the Anti-Inflammatory Effect of the MSC Secretome

To investigate whether the presence of anti-inflammatory drugs influences MSCs’ anti-inflammatory capacity, we evaluated the effect of their respective secretome on the in vitro NaIO_3_ damage-based inflammation model. This approach aimed to determine whether the exposure of MSCs to the tested drugs could modulate the immunomodulatory properties of the resulting secretome, potentially altering their ability to reduce inflammation. For this purpose, NO concentrations were measured in the culture supernatant using the Griess reagent, after exposing 10 mM NaIO_3_-damaged cells to the five secretomes produced in the presence of the different drugs.

**Figure 3:**
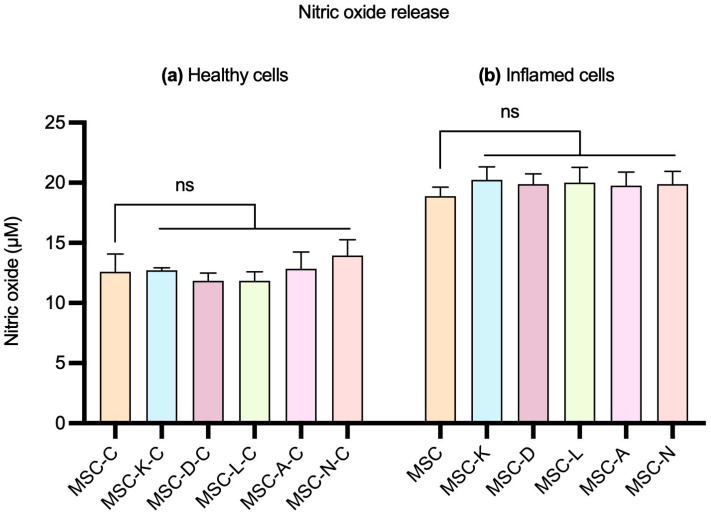
Effect of the control MSC secretome and MSC secretomes produced by the presence of anti-inflammatory drugs on NO release by ARPE-19 cells. (**a**) Healthy cells and (**b**) cells with 10 mM NaIO_3_-induced inflammation. MSC-C: no drug during secretome production; MSC-K: 100 µM ketorolac; MSC-d: 30.7 nM diclofenac; MSC-L: 100 µM α-lipoic acid; MSC-A 100 µM N-acetylcysteine; and MSC-N: 20 µM nicotinamide. No statistically significant differences in NO release were observed between control and inflammatory conditions in either healthy or inflamed cells. Data are shown as M (IQR). No statistically significant differences were observed. ns indicates no significant difference.

No statistically significant differences were observed in NO levels between ARPE-19 cells treated with secretomes produced by MSCs in the presence of drugs and by those treated with the control MSC secretome, in both healthy and inflamed ARPE-19 cells. In healthy cells, the M (IQR) of the NO levels was 12.59 (1.48) in the MSC-C group; 12.59 (0.37) in MSC-K-C; 11.48 (1.11) in MSC-D-C; 11.85 (1.48) in MSC-L-C; 12.22 (2.59) in MSC-A-C; and 14.07 (2.6) in MSC-N-C (Figure 3, left). In inflamed cells, the M (IQR) of the NO levels was 18.89 (1.48) in the MSC group; 19.63 (1.85) in MSC-K; 20.37 (1.48) in MSC-D; 19.26 (2.22) in MSC-L; 20 (2.22) in MSC-A; and 19.26 (1.85) in MSC-N (Figure 3, right) (*p* < 0.05).

### 3.3. The Presence of Anti-Inflammatory Drugs Does Not Alter BDNF or VEGF Levels in the MSC Secretome

To investigate whether the presence of anti-inflammatory drugs influences MSCs’ secretion of key regenerative molecules, the levels of BDNF and VEGF were quantified using specific ELISA assays. These two cytokines are well-recognized mediators of the therapeutic efficacy of the MSC secretome, playing essential roles in promoting cell survival, neuroprotection, angiogenesis, and tissue regeneration.

**Figure 4:**
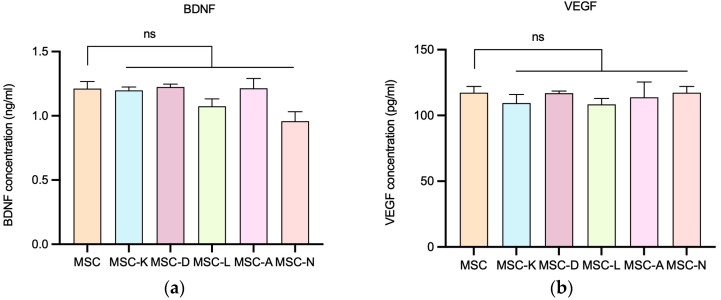
BDNF and VEGF secretion by MSCs following exposure to different secretomes produced in the presence of drugs. (**a**) BDNF concentration released by MSCs in the presence of drugs. (**b**) VEGF concentration released by MSCs in the presence of drugs. MSC-C: no drug during secretome production; MSC-K: 100 µM ketorolac; MSC-d: 30.7 nM diclofenac; MSC-L: 100 µM α-lipoic acid; MSC-A 100 µM N-acetylcysteine; and MSC-N: 20 µM nicotinamide. Data are shown as M (IQR). No statistically significant differences in BDNF or VEGF release were observed between the control and inflammatory conditions. ns indicates no significant difference.

No statistically significant differences were observed in secretome BDNF and VEGF concentrations between secretomes derived from MSCs in the presence of drugs and those from control MSCs in the presence of anti-inflammatory drugs. The M (IQR) of BDNF concentration (ng/mL) was 1.226 (0.102) in the MSC group; 1.200 (0.050) in MSC-K; 1.233 (0.038) in MSC-D; 1.078 (0.110) in MSC-L; 1.211 (0.147) in MSC-A; and 0.9655 (0.1411) in MSC-N (Figure 4a). The M (IQR) of VEGF concentration (ng/mL) was 206.2 (9.4) in the MSC group; 197.8 (12.7) in MSC-K; 205.4 (3.3) in MSC-D; 194.9 (7.7) in MSC-L; 198.1 (21.4) in MSC-A; and 204.1 (13.2) in MSC-N (Figure 4b). Concentration values were determined by extrapolating the absorbance measured in each sample using the equation derived from the kit’s standard curve (*p* < 0.05).

### 3.4. The Presence of Anti-Inflammatory Drugs Does Not Impair the Pro-Metabolic Effect of the MSC Secretome

To assess whether the presence of anti-inflammatory drugs influences MSCs’ secretome efficacy in promoting cell metabolic rate, we evaluated whether the presence of anti-inflammatory drugs induces changes in the MSC secretome’s capability to enhance the metabolic rate of the damaged cells. For this purpose, metabolic rate percentage was measured, after exposing 10 mM NaIO_3_-damaged cells to the five secretomes produced in the presence of the different drugs.

**Figure 5:**
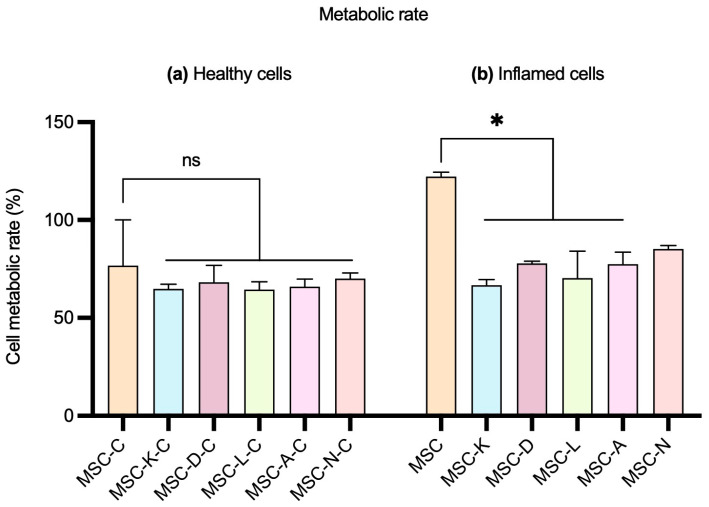
Effect of the MSC secretome on the metabolic rate of ARPE-19 cells. (**a**) Healthy ARPE-19 cells and (**b**) inflamed ARPE-19 cells with 10 mM of NaIO_3_. MSC-C: no drug during secretome production; MSC-K: 100 µM ketorolac; MSC-D: 30.7 nM diclofenac; MSC-L: 100 µM α-lipoic acid; MSC-A 100 µM N-acetylcysteine; and MSC-N: 20 µM nicotinamide. Data are shown as M (IQR). No statistically significant differences in cell metabolic rate were observed between control and inflammatory conditions in healthy cells. Significant differences were observed in inflamed cells between control and all but MSC-N conditions. * *p*-value < 0.05. ns indicates no significant difference.

No statistically significant differences were observed in cell metabolic rate between groups treated with secretome produced by MSCs in the presence of drugs and those treated with the control MSC secretome in healthy ARPE-19 cells. The M (IQR) values of cell metabolic rate (%) were 76.67 (38.89) in the MSC group; 65.56 (4.45) in MSC-K-C; 65.56 (16.67) in MSC-D-C; 63.33 (7.78) in MSC-L-C; 65.56 (7.78) in MSC-A-C; and 71.11 (5.55) in MSC-N-C (Figure 5, left). In inflamed ARPE-19 cells, a statistically significant decrease was observed when comparing cells treated with secretomes produced in the presence of drug to those treated with the control MSC secretome, except in the case of nicotinamide. The M (IQR) values of cell metabolic rate (%) were 122.2 (38.84) in the MSC group; 65.56 (5.56) in MSC-K; 77.78 (2.22) in MSC-D; 66.67 (26.67) in MSC-L; 74.44 (11.11) in MSC-A; and 85.56 (3.34) in MSC-N (Figure 5, right). However, cell metabolic rate maintained M-values over 65%. The percentage of metabolic rate was calculated by extrapolating the absorbance values obtained for each sample, using the control group as the 100% reference (*p* < 0.05).

## 4. Discussion

MSC-based therapies are gaining increasing importance in the fields of regenerative medicine and immunotherapy, due to their potent anti-inflammatory, immunomodulatory, anti-apoptotic, and pro-regenerative properties [25]. Comprising a diverse and complex mixture of cytokines, chemokines, growth factors, and extracellular vehicles, MSCs have shown significant potential in modulating immune responses, attenuating oxidative stress, and enhancing tissue repair mechanisms [55]. The present study was designed to explore how the presence of clinically relevant anti-inflammatory drugs might influence the composition and therapeutic efficacy of MSCs’ secretomes. As clinical interest in stem cell therapies continues to rise, particularly as a promising strategy against inflammatory diseases [56], it becomes increasingly important to understand how commonly used medications might interact with or alter the secretory behavior of these cells [57]. This consideration is particularly relevant in clinical scenarios where patients receiving MSC-based treatments are frequently on concurrent pharmacological regimens, including anti-inflammatory and antioxidant compounds that could alter the efficacy of MSC secretomes [58].

This study aimed to evaluate the effects of the presence of clinically relevant anti-inflammatory drugs on the secretory profile of MSCs, as well as their functional therapeutic properties. In the present study, AD-MSCs were selected, as they represent the most widely used source in clinical applications and are considered broadly representative, given that, as discussed previously, MSCs from different tissue origins exhibit largely comparable functional and paracrine properties. The drugs employed are relevant and directly involved in the inflammatory pathways, as detailed in the Introduction. Although a single in vitro inflammatory model was utilized, it is a well-established and extensively used system, supporting the representativeness and translational relevance of the observed findings.

Our results show that the functional efficacy of the MSC secretome was preserved, since its ability to exert an anti-inflammatory effect was not affected by the presence of any of the five anti-inflammatory drugs, supporting the possibility of delivering a drug–secretome combined treatment. These findings align with a growing body of literature examining the effects of the presence of anti-inflammatory drugs on MSCs [59,60,61]. Most studies have shown that treatment with various drugs does not impair the phenotype, viability, or core therapeutic functions of MSCs [62,63], and, in several cases, pharmacological conditioning has been reported to enhance the anti-inflammatory and regenerative potential of the MSC secretome [64,65]. Analyzing the individual molecules of the secretome, we observe that MSCs’ secretion level of two critical trophic factors—BDNF and VEGF—remained unaltered in the presence of five different anti-inflammatory drugs.

Most importantly, although significantly compromised in the presence of four of the five drugs tested in the study, the cell metabolic rate was maintained at over 65% values when treated with secretomes produced in the presence of anti-inflammatory drugs.

Collectively, these findings support the robustness of MSCs as a therapeutic tool in inflammatory contexts and suggest that pre-exposure to selected drugs does not compromise their functional efficacy, secretory profile, or anti-inflammatory properties. This insight is critical for the potential clinical application of MSC-based therapies, particularly in settings where patients may be receiving concurrent drug treatments. Although only a single MSC type was employed, as discussed in previous responses, this does not constitute a major limitation, given that MSCs from different sources exhibit largely comparable properties. Regarding the inflammatory model, it is a well-established and widely used system in experimental studies and is therefore considered representative of inflammatory responses. Nevertheless, for clinical translation, it would be important to evaluate additional models to confirm whether the observed effects are consistent, as well as subsequently extending these findings to in vivo settings.

## 5. Conclusions

The primary conclusion of this study is that the exposure of MSCs to the tested pharmacological agents does not significantly alter their BDNF or VEGF content, nor does it impact their anti-inflammatory or pro-metabolic activities within the employed inflammatory model.

Exposure to specific pharmacological agents can be compatible with, or even beneficial for, maintaining and potentially augmenting the functional properties of MSCs without compromising their therapeutic efficacy [66,67]. However, the decrease in cellular metabolic activity in treatment with secretomes produced in the presence of the anti-inflammatory drugs tested in this study should be taken into account in order to decide upon the convenience of a combined treatment; the effect of more drugs should be performed.

By clarifying the impact of clinically relevant anti-inflammatory drugs on MSC-derived secretomes, this work offers critical insight into the design of robust and compatible MSC-based therapies, particularly in the context of combinatory treatments for inflammatory diseases.

While this work provides valuable findings, certain aspects could be further explored in future research. Expanding the panel of drugs may offer a broader understanding of the drug-induced modulation of the MSC secretome. The cell damage model employed could be applied to other cell lines; nevertheless, incorporating additional models, including primary human cells, would strengthen translational relevance. Finally, although two key secretome components (BDNF and VEGF) were analyzed, extending the molecular profiling could yield a more comprehensive view of the effects observed.

## Data Availability

Data will be available upon request.

## Abbreviations

The following abbreviations are used in this manuscript:

MSCs	Mesenchymal stem cells
BDNF	Brain-Derived Neurotrophic Factor
VEGF	Vascular Endothelial Growth Factor
NaIO_3_	Sodium iodate
ARPE-19	Linear dichroism
NO	Nitric oxide
M (IQR)	Median with interquartile range
ANOVA	Analysis of Variance
